# Accuracy of Citrate Anticoagulant Amount, Volume, and Concentration in Evacuated Blood Collection Tubes Evaluated with UV Molecular Absorption Spectrometry on a Purified Water Model

**DOI:** 10.3390/molecules28020486

**Published:** 2023-01-04

**Authors:** Nataša Gros, Tadej Klobučar, Klara Gaber

**Affiliations:** Faculty of Chemistry and Chemical Technology, University of Ljubljana, Večna Pot 113, SI1000 Ljubljana, Slovenia

**Keywords:** blood collection tubes, citrate anticoagulant, direct spectrometric determination, quality control method, anticoagulant concentration, draw volume, anticoagulant volume, magnesium contamination, potassium contamination

## Abstract

Citrate anticoagulant concentration affects the results of coagulation tests. Until now, the end user had no direct insight into the quality of evacuated blood collection tubes. By introducing an easy-to-perform UV spectrometric method for citrate determination on a purified water model, we enabled the evaluation of (1) the accuracy of the anticoagulant amount added into the tubes by a producer, (2) the accuracy of the volume of anticoagulant solution in the tube at the instant of examination, (3) the anticoagulant concentrations at a draw volume. We examined the Vacuette^®^, Greiner BIO-ONE, Vacutube, LT Burnik d.o.o., and BD Vacutainer^®^ tubes. The anticoagulant amount added into the tubes during production had a relative bias between 3.2 and 23.0%. The anticoagulant volume deficiency at the instant of examination expressed as a relative bias ranged between −11.6 and −91.1%. The anticoagulant concentration relative bias after the addition of purified water in a volume that equalled a nominal draw volume extended from 9.3 to 25.7%. Draw-volume was mostly compliant during shelf life. Only Vacutube lost water over time. Contamination with potassium, magnesium, or both was observed in all the tubes but did not exceed a 0.21 mmol/L level. This study enables medical laboratories to gain insight into the characteristics of the citrate blood collection tubes as one of the preanalytical variables. In situations that require anticoagulant adjustment for accurate results, this can help make the right decisions. The methodology gives producers additional means of controlling the quality of their production process.

## 1. Introduction

Specimen collection, transport, storage, and processing are widely recognised preanalytical variables in coagulation testing [1]. Verification and validation protocols of blood collection tubes of different brands or lots assume a comparison to be performed on blood samples [2,3]. No method providing direct insight into the quality of the citrate anticoagulant blood collection tubes has been reported yet.

In the past, when anticoagulant solution was introduced into blood collection tubes in medical laboratories, they were in full control of this preanalytical phase which only required an accurately measured volume of a stock anticoagulant standard solution of accurate concentration and an accurate specimen volume measurement. The COVID-19 crisis caused a shortage of commercial tubes and temporarily revived this approach and its straightforwardness [4].

The introduction of evacuated blood collection tubes in the 1940s simplified phlebotomy and improved personnel safety [5]. Even though the tubes are easy to use, they are complex blood collection devices. Several previously unknown problems emerged, e.g., the material’s water- and air-tightness, hydrophobicity, draw-volume accuracy, and additives as potential sources of specimen contamination [6,7].

Glass and plastic evacuated tubes were compared for coagulation tests, and although some statistically significant differences were confirmed, they were not recognised as being of clinical importance [8,9,10,11,12]. The 109 mmol/L citrate concentration was assumed to better support platelet aggregation than the 129 mmol/L citrate solution, and the advantages of buffered citrate were not recognised [13].

With the evacuated blood collection tubes, specimen volume is defined by the tube’s draw capacity. It was suggested that routine coagulation tests had to be validated for minimal citrate tube fill volume to ensure accurate results [14]. The effect of the concentration of trisodium citrate anticoagulant on a calculation of the international normalised ratio and the international sensitivity index of thromboplastin was confirmed [15]. Prolonged prothrombin time and activated partial thromboplastin time due to underfilled specimen tubes were observed [16], and for patients with high haematocrit values, citrate concentration must be adjusted for accurate results [17]. Studies assessing thrombin generation are sensitive to a change in tube brand, and the choice of draw volume is important since low draw volumes can bias results [18].

On one side, the quality of blood-collection devices is considered a producer responsibility [19], but on the other, medical laboratories are fully responsible for the results of coagulation tests. With no direct insight into the characteristics of the citrate anticoagulant blood collection tubes, they remain unknown, which is difficult to control. Several studies confirmed that citrate concentration in a specimen affects the results of coagulation tests, and differences between brands, and even lots, were observed.

Evacuated citrate anticoagulant blood collection tubes are supposed to ensure the dilution of a specimen with the anticoagulant solution in a proportion of nine to one. To reach a correct anticoagulant level in a specimen, not only the draw volume but also the anticoagulant amount introduced into a tube during production and the volume of anticoagulant solution at the instant of venepuncture should be accurate. Anticoagulant volume accurately measured in the production setting changes during the tube evacuation process because of water evaporation. Consequently, the anticoagulant solution concentration increases.

A methodology providing direct insight into the quality of citrate anticoagulant tubes is required to properly regulate the anticoagulant volume and concentration during production and to enable medical laboratories to control the tubes as an input variable of their analytical process. No such methodology has been reported yet.

The titrimetric method suggested by the standard [20] is only suitable for the quality control of a bulk anticoagulant solution before it enters the production process. Conductometry, by which we enabled quality evaluation of K2EDTA and K3EDTA tubes [21,22], is not applicable since buffered and unbuffered citrate differ in chemical equilibrium forms [23,24]. In addition to the appropriate analytical method, the upgraded methodology is necessary since citrate tubes have several additional sources of uncertainty if compared to EDTA tubes.

The first objective of our work is to develop a low-cost, easy-to-apply methodology that would, by using purified water as a model, enable the evaluation of the following:

- the accuracy of the anticoagulant amount added into the tubes by a producer;
- the accuracy of the volume of anticoagulant solution in the tube at the instant of examination;
- the anticoagulant concentrations corresponding to the nominal draw or draw volume.

The second objective is to provide insight into the quality of the tubes of three different brands, namely Vacuette^®^—Greiner BIO-ONE, Vacutube—LT Burnik d.o.o., and BD—Vacutainer^®^ that would involve the above-listed parameters together with water loss over time, draw volume during shelf life, and contamination examination.

## 2. Results

Citrate species absorb UV light, and spectra are influenced by chemical equilibrium. Krukowski et al., by lowering the pH of the solution below 1, shifted chemical equilibria towards the entirely protonated form and enabled citrate determination in oral electrolyte formulations [25]. To enable the quality evaluation of blood collection tubes, we adapted the method of Krukowski et al. In Section 2.1, we confirm the method’s performance and fitness for the purpose. In Section 2.2, we present the results of the quality evaluation of citrate anticoagulant blood collection tubes before their intended use for specimen collection performed on a model of purified water. Water loss during the time, draw volume changes during shelf life, the accuracy of the amount of the anticoagulant added into a tube by a producer, and the accuracy of the volume of the anticoagulant solution in a tube at the instant of the examination are studied.

### 2.1. Spectrometric Method for Citrate or Buffered Citrate Determination

#### 2.1.1. Method Performance

**Figure 1:**
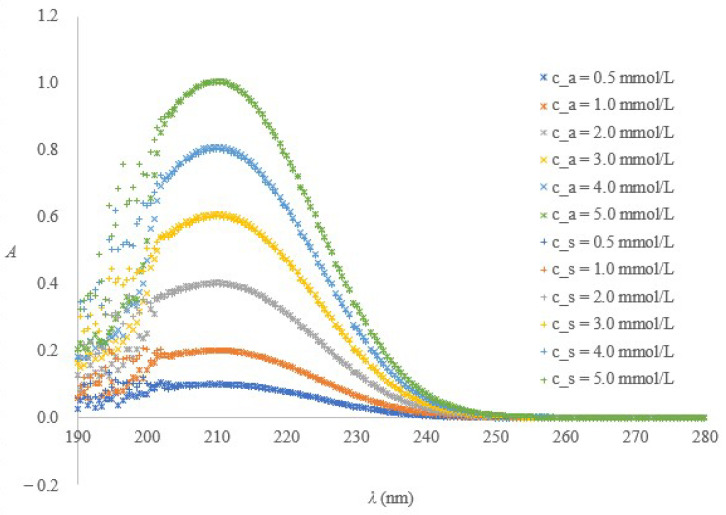
Citrate and citric acid model solutions-based confirmation that the suggested spectrometric method, independently of the initial equilibrium forms, ensures identical absorption spectra (c_a and c_s denote the citric acid and trisodium citrate amount concentration).

The spectra obtained for trisodium citrate or citric acid solutions prepared in HCl medium proved identical at all the examined concentration levels. Figure 1 confirms that the HCl addition is adequate to efficiently shift the citrate chemical equilibrium towards the entirely protonated citrate form. The method is consequently equally well suited for the evaluation of trisodium or buffered citrate solutions.

A single factor ANOVA with concentration as a controlled factor applied to the absorbance ratios obtained at 210 nm vs. 230 nm wavelengths during a month proved no significant between-groups differences for citrate or for citric acid solutions. The *F*_5/30_(*p* = 0.05) and *F* calculated for citrate were 2.534 and 0.7831. The *F*_5/36_(*p* = 0.05) and *F* calculated for citric acid were 2.477 and 1.884. Since the calculated *F* was lower for the former, citrate was recognised as a better choice for the preparation of calibration solutions. The 210 nm wavelength was selected for the absorbance measurements. A visual inspection of six-point calibration graphs confirmed the linearity within the targeted citrate concentration range extending from 0.25 to 4 mmol/L.

The within-day repeatability of the spectrometric procedure was tested on the 14.12 mmol/L trisodium citrate stock standard solution. The relative standard deviation of the determined citrate concentration was 0.18%, and the bias expressed relatively was 0.3%. Absorbances measured in a 1-cm cuvette at 210 nm against the HCl blank and the determined citrate concentrations for 10 replicates are given in Table 1. The symbol *s* denotes a standard deviation of a statistical sample.

.

Data in Table 2 verify the within-laboratory reproducibility of calibration parameters for a six-month period. A slope *a*, a standard deviation of the slope *s_a_*, an intercept *b*, a standard deviation of the intercept *s_b_*, a standard error of the estimate *s_y_*_/*x*_, and a coefficient of determination *R*^2^ are given.

In Table 3, minimum and maximum citrate concentrations determined on the Milli-Q water model for the blood collection tubes examined on a particular day are stated together with their combined standard uncertainties. The major source of uncertainty was the standard uncertainty of interpolation of concentration *x*_0_ from the calibration line equation. It is denoted by *s_x_*_0_ and evaluated by applying Equation (1). The symbols $x_{i}$, *y*_0_, $\bar{x}$, and $\bar{y}$, not explained previously, stand for the concentrations of the citrate calibration solutions, the absorbance of the examined solution, and the coordinates of the centroid of the calibration line equation, respectively.

(1) $$s_{x_{0}}=\frac{s_{y/x}}{a}\sqrt{1+\frac{1}{n}+\frac{{\left(y_{0}-\bar{y}\right)}^{2}}{a^{2}\sum {\left(x_{i}-\bar{x}\right)}^{2}}}$$

The relative standard uncertainties of interpolation, which varied between 0.052 and 0.0054 depending on the day of the experiment, were combined with the 0.0087 relative standard uncertainty of the 10-fold dilution required by the spectrometric procedure. Other uncertainty contributions associated with the spectrometric procedure proved negligible. The derived combined standard uncertainties *u*_c_ vary between 0.1 and 0.7 mmol/L (Table 3).

#### 2.1.2. Fitness for Purpose

**Figure 2:**
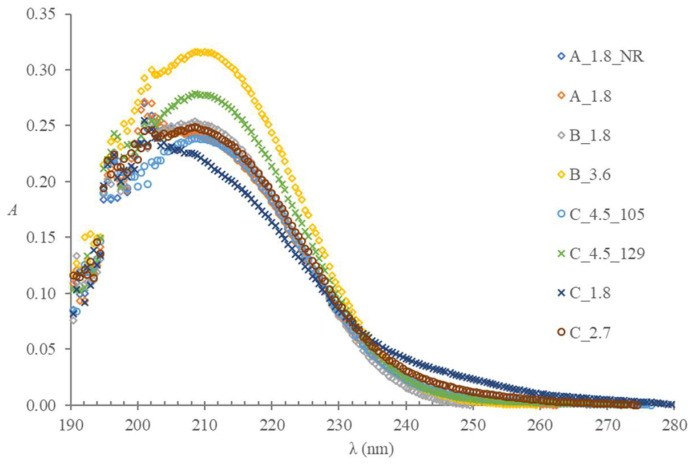
The spectra of citrate or buffered citrate solutions obtained from the tubes of different producers prepared in HCl medium and recorded in a 1-cm cuvette against the HCl blank.

In the wavelength range relevant to the method, the spectra of the citrate or buffered citrate solutions obtained from the tubes of different producers (Figure 2) prepared in the HCl medium and recorded in a 1-cm cuvette against the HCl blank proved very similar to the spectra of the calibration solutions (Figure 1). The only exception is a spectrum of the solution extracted from the C 1.8 tubes, which has a distinct shape within the 200 to 230 wavelength range. The letters A, B, and C denote producers, the number that follows indicates a nominal draw volume, NR means non-ridged, and numbers 105 and 129 distinguish the tubes with less common anticoagulant concentration from the rest with the 109 mmol/L concentration. More details are given in Section 4.2.

**Figure 3:**
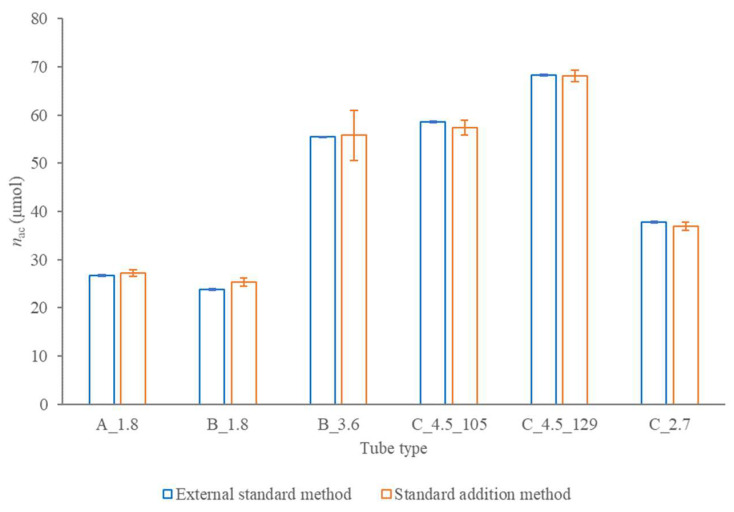
Anticoagulant amount per tube with 95% confidence interval determined in composite samples with the external standards (blue columns) and standard additions (orange columns) calibration method.

Figure 3 confirms no apparent interference effect for the examined tube types since the anticoagulant amount expressed per a single tube and determined in composite samples proved comparable for the external standard and standard addition calibration method.

The spectrometric method proved reliable for the trisodium or buffered citrate concentration determination in blood collection tubes. Even though the results for C_1.8 tubes are not beyond any doubt, the inspection of the shape of several spectra implies that the absorbance changes more profoundly below 210 nm.

### 2.2. Evacuated Blood Collection Tubes for Coagulation Tests—Quality Evaluation

Quality evaluation of blood collection tubes comprises water loss during the time (Section 2.2.1), draw volume changes during shelf life (Section 2.2.2), the accuracy of the amount of the anticoagulant added into a tube by a producer (Section 2.2.3), and accuracy of the volume of the anticoagulant solution in a tube at the instant of the examination (Section 2.2.4).

#### 2.2.1. Water Loss during the Time

We used one-factor ANOVA to test the hypothesis that water can evaporate from the blood collection tubes over time. The control factor was time and the examined variable was the mass of the tubes. The test was applied to the tubes of all three producers marked as already explained but extended with the expiration date. For the tubes A_1.8_9.7., C_4.5_31.7., and C_4.5_31.12. the hypothesis was not confirmed. For tubes B_1.8_31.7. and B_3.6_31.8. time proved a significant factor. The values 50.93 and 44.06 of the calculated *F* of the two types of tubes highly exceeded the critical value of 2.39 at the 0.05 significance level. The lowest significant differences, *LSD*, calculated by Equation (2) were 0.01916 and 0.02038, respectively. Symbols *t*, *s*, *h*, and n stand for the Student’s *t* factor, within-sample standard deviation, number of groups, and number of within-group repetitions. Significant differences are indicated by asterisks in Table 4.

(2) $$LSD=t_{h\;\left(n-1\right)}\cdot s\cdot \sqrt{2/n}$$

#### 2.2.2. Draw Volume during the Time

**Figure 4:**
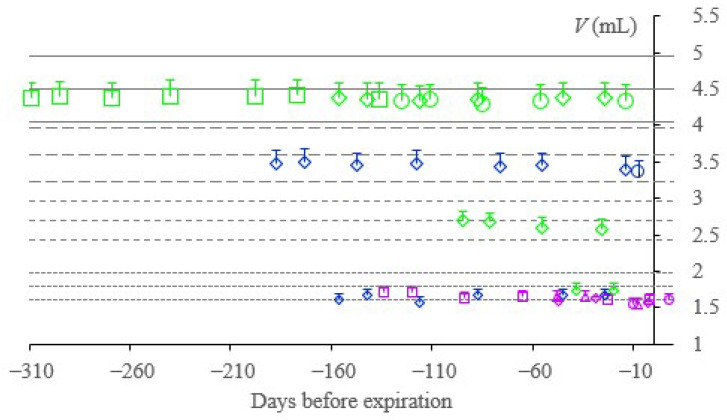
Draw volumes determined in Ljubljana at 300 m altitude, at ambient temperature during a life-cycle of the tubes; the error bars indicate the draw volume as assumed to be at 1013 hPa and 20 °C; the violet, blue, and green markers pertain to the tubes A, B, and C; the horizontal lines of a particular type define the 10% range around the target draw volume.

Figure 4 presents the draw volumes determined with a 5-mL Bang burette in Ljubljana at 300 m altitude at ambient temperature for the tubes of the three producers during their life cycle. Tubes A, B, and C were assigned markers of violet, blue, and green colour, respectively. The markers of the tubes are graded in size regarding their nominal draw volume, difference in shape indicate different lots or brands. The horizontal lines of a particular type define the 10% range around the targeted draw volume.

#### 2.2.3. Accuracy of the Amount of the Anticoagulant Added into a Tube by a Producer

Table 5 presents the expected anticoagulant amount per tube (*n*_ac_expt_), which is derived from the nominal anticoagulant concentration (*c*_ac_nom_), together with the determined amount (*n*_ac_dtmn_). The discrepancy is expressed as relative bias. The anticoagulant concentration that would have been expected in the tube (*c*__*V*_total_nom_), if both the nominal anticoagulant volume (*V*_ac_nom_) and the nominal draw volume (*V*__draw_nom_) hold, is calculated.

#### 2.2.4. Accuracy of the Volume of the Anticoagulant Solution in a Tube at the Instant of the Examination

Anticoagulant concentration (*c*_ac_dtmn_) determined in a tube after the addition of Milli-Q water into a blood-collection tube in a volume that equals *V*__draw_nom_ is given in Table 6. An anticoagulant volume that was present in a tube at the instant of the experiment (*V*_ac_) was calculated. The relative bias of both parameters is given.

### 2.3. Contaminants or Potential Additives

Table 7 summarises the potassium and magnesium concentrations, *c* (K^+^) and *c* (Mg^2+^), respectively, determined with atomic absorption spectrometry in composite samples prepared with Milli-Q water from several tubes of the same type. Concentrations are expressed so that they represent potassium and magnesium content after a draw if *V*__draw_nom_ and *V*_ac_nom_ comply with the declaration.

## 3. Discussion

### 3.1. What Makes the Quality Evaluation of the Citrate Blood Collection Tubes More Challenging If Compared to the EDTA Tubes

In 2013, we conducted quality control of EDTA anticoagulant evacuated blood collection tubes before specimen collection by developing a methodology that can be summarised in three points [21].

(a) Conductometry was confirmed as an adequate low cost easy-to-perform analytical method for the determination of EDTA anticoagulant concentration in tubes on a deionised or distilled water model.
(b) A 5-mL-Bang burette-based method for the determination of draw volume on a model of deionised or distilled water in the laboratory setting at local experimental conditions was established.
(c) A physical model was developed, which by correcting the measurements for the ambient temperature and the unreduced air pressure influence at the altitude of the experimental setting above sea level, was able to predict a draw volume and blood anticoagulant concentration for specimen collection under the assumption of no other phlebotomy-related adverse effect.

The necessary input variables are only the tube internal volume (*V*_int_), the temperature, and unreduced air pressure at the time of the laboratory experiment.

The methodology, which we applied to the Becton Dickinson, Greiner Bio-One, and Laboratorijska tehnika Burnik d.o.o. evacuated blood-collection tubes, revealed that draw-volume deficiencies are relatively rare. Noncompliance with the H1-A5 standard [20] regarding exceeding EDTA anticoagulant concentrations was much more frequent. We wished for a similar methodology to be developed for citrate tubes but recognised them as a distinct and much more challenging case [21,22].

**Figure 5:**
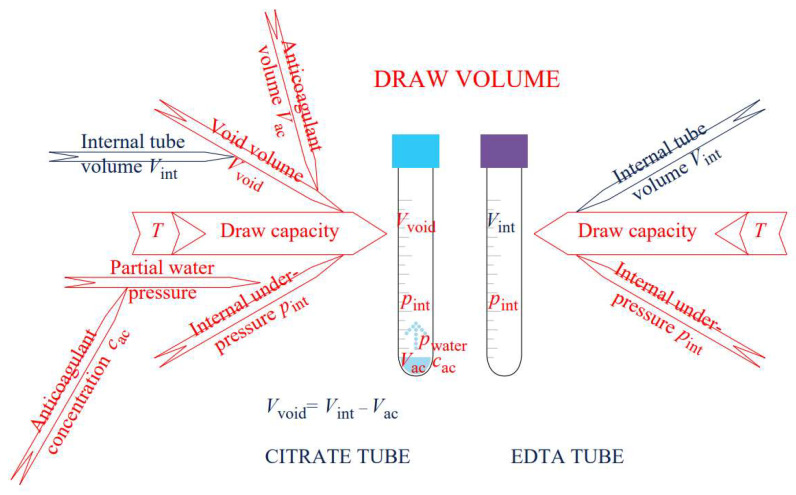
Parameters influencing a draw volume in the citrate (**left**) and EDTA (**right**) blood collection tubes, parameters that vary are indicated in red.

As Figure 5 illustrates, a draw capacity of an EDTA tube depends only on internal under-pressure (*p*_int_), ambient temperature, and *V*_int_, which can easily be determined. The physical model for correcting the draw-volume measurements obtained under ambient conditions fully applies. Consequently, the concentration of anticoagulant can be predicted for specimen collection. Anticoagulant, if not present in a dry form, has a negligibly small volume in comparison with *V*_int_. A study of the change in the draw capacity of the tubes of different producers during the time was possible [22].

In contrast, *V*_int_ does not define the draw capacity of citrate tubes; since *V*_ac_ occupies part of *V*_int_, the latter does not equal the void volume, *V*_void_. The *V*_ac_ is an unknown, and the value declared by the producer does not necessarily hold, it can decrease over time due to water evaporation, and it is not directly determinable by simple means. Consequently, *V*_void_ is unknown, too. The parameter *p*_water_, which is temperature and *c*_ac_ dependent, affects *p*_int_. Determination of *c*_ac_ is yet another problem. For the majority of analytical methods, especially low-cost ones, *V*_ac_ is too small, and if it changes during time, *c*_ac_ changes consequently.

We had to find a way around these problems. By adding Milli-Q water in a volume that equals the *V*__draw_nom_, one does not directly get insight into the correctness of the anticoagulant amount, *n*_ac_ introduced into the tube at the instant of production since the total solution volume is unknown due to dilution of *V*__draw_nom_ by the unknown *V*_ac_. By not knowing *n*_ac_ and *V*_ac_, one cannot evaluate the correctness of the anticoagulant concentration for specimen collection. In the continuation, we explain the methodology for obtaining insight into *n*_ac_ and *V*_ac_.

### 3.2. Quality of Evacuated Blood Collection Tubes for Coagulation Tests

The results of the citrate determination in the composite samples (*c*_dtmn_comp_) provide insight into the correctness of the anticoagulant amount (*n*_ac_dtmn_) added into the tubes during production (Table 5). The amount was calculated by Equation (3). *V_f_* and *N* stand for the volumetric flask volume and the number of tubes used for the preparation of the composite sample, respectively.

(3) $$n_{ac\_dtmn}=\frac{c_{dtmn\_comp}\cdot \;V_{f}}{N}$$

All the anticoagulant amounts exceed the producers’ declarations. All three sets of the C_4.5 tubes, distinguished by their expiration dates, have the anticoagulant amount the closest to the expected value. The bias was between 3.2 and 6.9%. The tubes B_1.8 and B_3.6, by not exceeding the 10% tolerance limit, can still be considered in accordance with the standard [20]. Except for the already mentioned tube B_1.8, the rest of the tubes with 1.8 mL paediatric draw, namely A_1.8 with two distinct expiration dates and tubes C_1.8, exceed the expected citrate amount by more than 20%. The results of the latter might be overestimated due to the irregularities observed in the absorption spectrum, indicating the possible contribution of an unidentified additive or contaminant. The tubes C_2.7 are also non-compliant, with the citrate amount exceeding the declared value by 17.4%.

The tubes exposed here as potentially compliant would have been such only if the anticoagulant volume and the draw volume had been correct, too. The amount of citrate in the tubes (*n*_ac_dtmn_, Table 5) together with the citrate determination (*c*_dtmn_, Table 6) after the addition of Milli-Q water into the tubes in the volume corresponding to *V*_draw_nom_ enabled us to judge the correctness of the anticoagulant volume (*V*_ac_) in the tubes at the instant of the examination (Table 6). Equation (4) was applied. For the sake of clarity, *c*_dtmn_ is in the equation indicated as *c*_nom_draw_*V*_.

(4) $$V_{ac}=\frac{n_{ac\_dtmn}-V_{\_draw\_nom}\;\cdot \;c_{nom\_draw\_V}}{c_{nom\_draw\_V}}$$

Water loss is material-dependent [7]. Even though the experimental data in Table 4 confirmed that tubes B lost water over time, tubes B_3.6 had the most accurate anticoagulant volume of all the examined tubes, with a bias of −17% (Table 6). The explanation is that the water loss experiment started five months before the expiration date and the first two dates of weighing the tubes were the 25th of February and the 11th of March. The evaluation of the correctness of the anticoagulant volume followed soon after, on the 21st of March. Water loss, even though recognised as statistically significant, could not have been yet very high in comparison with the target 0.4 mL anticoagulant volume. The 129 mmol/L target anticoagulant concentration also contributed by decreasing the partial water pressure. The impact on the B_1.8 tube with an expiration date only a month sooner but with the declared anticoagulant concentration of 109 mmol/L was entirely different. The anticoagulant volume bias was −91%, very similar to the C_1.8, 109 mmol/L tubes (−96%), and C_4.5_31.7., 105 mmol/L tubes (−90%). The anticoagulant volume bias was lower for the remaining tubes of producer C and ranged from −73% to −45%. The tubes A_1.8_9.4. and A 1.8_9.7. are by the anticoagulant volume bias −33% and −22% of a distinctively better quality regarding the already discussed tubes of the producers B and C with the paediatric draw.

To make a synthesis of the results of the two experiments, we calculated the concentrations *c*__*V*_total_nom_ (Equation (5)) that could have been expected for the determined anticoagulant amount if the assumption that both *V*__draw_nom_ and *V*_ac_nom_ hold (Table 5). The *V*__total_nom_ stands for the total nominal volume.

(5) $$c_{\_V\_total\_nom}=\frac{n_{ac\_dtmn}}{V_{total\_nom}}=\frac{n_{ac\_dtmn}}{V_{draw\_nom}+V_{ac\_nom}}$$

**Figure 6:**
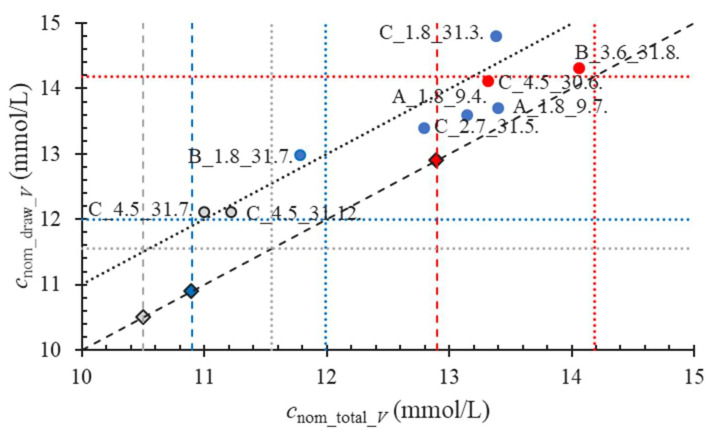
Relation between *c*__V_total_nom_ and the *c*_nom_draw_*V*_. The grey, blue, and red dots pertain to the tubes with 105, 109, and 129 mmol/L nominal anticoagulant concentrations, respectively. The diamonds in the same colours and the vertical dashed lines represent the corresponding accurate concentrations. The grey, blue, and red dotted vertical lines are the 10% upper limits of the anticoagulant concentrations. The dashed diagonal line is the line with a slope of 1.

In Figure 6, we relate *c*__V_total_nom_ with the *c*_nom_draw_*V*_. The grey, blue, and red dots pertain to the tubes with 105, 109, and 129 mmol/L nominal anticoagulant concentrations, respectively. The tube labels are indicated. The diamonds in the same colours and the vertical dashed lines represent the corresponding accurate concentrations. The grey, blue, and red dotted vertical lines are the 10% upper limits of the anticoagulant concentrations. The dashed diagonal line is the line with a slope of 1.

Since the grey dots pertaining to the C_4.5_31.7. and C_4.5_31.12. 105-mmol/L anticoagulant tubes both lay within the grey vertical lines, the anticoagulant amount is within the 10% acceptance limit. The former set of tubes, by crossing the dotted diagonal line, exhibit more than 90% anticoagulant volume deficiency, the latter a 73% deficiency. Even with the accurate draw volume, the anticoagulant concentration would, with 12.1 mmol/L, exceed the 10% upper acceptance anticoagulant concentration limit, presented by the grey horizontal line.

The red dots correspond to the 129 mmol/L tubes C_4.5_30.6. and B_3.6_31.8. Both have anticoagulant amounts within the acceptance limits. The former of all the tubes is the only one entirely compliant with the standard [20]. The later dot laying the closest to the dashed diagonal line has the lowest anticoagulant volume deficiency of all the tubes. Since the water loss during the time was confirmed for this type of tube, the dot will be moving during the time towards the dotted diagonal line. The anticoagulant concentration, which at the instant of the experiment for the correct draw volume only slightly exceeded the upper anticoagulant limit, would rise.

Among the blue dots representing the 109 mmol/L tubes, only the B_1.8_31.7. tubes have an anticoagulant amount within the acceptance range, but their anticoagulant volume deficiency exceeded 90% since the tubes are not watertight. Consequently, the anticoagulant concentration for the nominal draw volume highly exceeds the dotted horizontal blue line. On the contrary, the dots A_1.8_9.4. and A_1.8_9.7, by being quite close to the dashed diagonal line, exhibit the lower anticoagulant volume deficiency of all the tubes with a paediatric draw, but the anticoagulant concentration at the nominal draw volume highly exceeds the 10% concentration limit, since the amount of anticoagulant in the tube is too high.

As we previously observed in EDTA tubes, citrate tubes also mostly have a draw volume within the 10% tolerance limit (Figure 4). The upper tolerance bar end is relevant for specimen collection. We did not correct the draw-volume measurements by applying the physical model due to already-explained limitations. Regarding our previous experiences, approximately a 5% increase in draw volume compensates for the altitude of our experimental setting. Lippi et al. also confirmed the adequate quality of the citrate tubes regarding their draw volume [26], and new materials are promising to limit the time-dependent decrease in draw volume [27], which we observed.

### 3.3. Contaminants or Potential Additives

UV absorbance spectra of the citrate anticoagulant extracted with Milli-Q water from the C_1.8, C_2.7, and C_4.5 tubes, prepared in HCl medium, and recorded against the HCl blank in 1-cm cuvette indicate possible additives absorbing in the wavelength range between 235 and 265 nm (Figure 2).

These were also the only tubes in which we were able to determine potassium with atomic absorption spectrometry. The *c*__*V*_total_nom_ ranged from 139 to 170 μmol/L (Table 7). Even though not successfully confirmed with IR spectra due to high citrate concentration, one of the possible assumptions would be cross-contamination with K_3_EDTA or K_2_EDTA in a production setting. Lima-Oliveira et al., by simulating cross-contamination of citrate tubes with EDTA during phlebotomy, confirmed that 29% of K2EDTA blood causes a significant bias in the results of routine clotting assays [28]. Concentrations originating from the tube contamination, which we observed, are far below this limit.

The tubes C_1.8 raise additional concerns about other unidentified additives or contaminants absorbing in the UV range (Figure 2). No tubes were entirely free of contamination with metallic ions, as Table 7 demonstrates. Tubes A and B were contaminated with magnesium ions, tubes C_1.8 and C_2.7 with potassium ions, and tubes C_4.5 with potassium and magnesium ions.

Van der Besselaar et al. recognized a tube-stopper as a possible source of magnesium contamination influencing the prothrombin time [29]. They suggested a 1 mmol/L level as the maximal admissible concentration [30] and confirmed that low-magnesium tubes fulfil this requirement [31]. We confirmed magnesium contamination in the tubes of all three producers, but no concentration would exceed 210 μmol/L if the total solution volume corresponds to the sum of the nominal anticoagulant solution volume and nominal draw volume (Table 7). It should be mentioned that the results only reflect contamination of the inherent citrate anticoagulant solution, the volume of which is small. Determination of metallic anions was performed in composite samples prepared, as the last paragraph of Section 4.1 describes. The procedure did not involve a within-tube solution mixing. With a stopper as a source of contamination, higher concentrations are likely in such cases.

### 3.4. Implications and Limitations

The suggested methodology is easy to apply. It does not require any special costly instrumentation. Spectrometers for molecular absorption spectrometry are widely available. If the medical laboratory only has dedicated equipment, every university with natural science study programs certainly has a general-purpose spectrometer. Compact, low-cost spectrometers are available at affordable prices. The quality of the tubes entering the analytical process can be directly evaluated on a distilled or deionised water model. With the methodology, we suggest it is possible to predict the anticoagulant concentration for specimen collection, assuming no phlebotomy-related adverse effects. One only needs to replace *V*__draw_nom_ in Equation (5) with a measured draw volume. Further methodology development is possible. Irregularities in absorption spectra observed for 1.8-mL tubes from producer C need clarification.

The suggested methodology gives a final user a direct insight into the tubes’ quality and provides the tube producers with additional means of controlling the quality of their products.

## 4. Materials and Methods

### 4.1. Spectrometric Method for Citrate or Buffered Citrate Determination

Spectrometric measurements were performed with a Varian Cary 50 UV-Vis spectrometer, Agilent Technologies, Santa Clara, CA, USA in a 1-cm cuvette against 126 mmol/L HCl solution. Absorbance was measured at 210 nm.

Deionised water was additionally purified through the Milli-Q system (Millipore, Billerica, MA, USA)—Milli-Q water in the continuation and A-class glass volumetric equipment were used for the preparation of solutions if not stated differently.

Six stock calibration solutions were prepared in 100-mL or 200-mL volumetric flasks by weighing trisodium citrate dihydrate C_6_H_5_Na_3_O_7_ ∙ 2H_2_O (*M* = 294.10 g/mol, w ≥ 0.98), CAS: 6132-04-3, Sigma-Aldrich, St. Louis, MO, USA. The diluent was Milli-Q water. Concentrations extended from 2.5 to 40 mmol/L in the final method.

Each working calibration solution was prepared in a 50-mL volumetric flask by measuring 5 mL of its corresponding stock solution with the glass volumetric pipette. The solution was made up to the mark with the 140 mmol/L HCl, prepared from the 37% HCl (CAS: 7647-01-0, *ρ* = 1.19 g/mL, Honeywell, Seelze, Germany).

For testing the repeatability of interpolation of citrate concentration from the calibration line equation, 14.12 mmol/L citrate stock solution was prepared in a 50-mL volumetric flask. Ten testing solutions were prepared from it by following the procedure as described in the previous paragraph.

For citrate determination in the evacuated blood collection tubes, the tubes were filled with Milli-Q water. The methods of filling were goal dependent. The draw volume method is described in Section 4.2.2. For the addition of a nominal volume, a Milli-Q water volume was measured with a 5-mL Bang burette directly into uncapped tubes. In both cases, solution was mixed as the producer suggested, and a 1-mL aliquot was transferred into a 10-mL volumetric flask with the A class Normax glass volumetric pipette, Duran, Portugal, and diluted with the 140 mmol/L HCl to the mark.

For citrate determination in the composite sample, the citrate or buffered citrate was quantitatively transferred from the required number of tubes of a particular type into a 50-mL volumetric flask with Milli-Q water. The solution was made up with Milli-Q water to the flask’s nominal volume and further treated as the UV spectrometric method or atomic absorption spectrometry required.

### 4.2. Evacuated Blood Collection Tubes

The evacuated blood collection tubes were purchased from the local Slovene dealers. Two brands were global and one local. They were assigned letters A—Vacuette^®^, Greiner BIO-ONE, B—Vacutube, LT Burnik d.o.o., Skaručna, Slovenia, and C—BD Vacutainer^®^. The abbreviations, main features, and expiration dates are summarized in Table 8.

#### 4.2.1. Water Loss during a Time

The experiment was repeated for four months at regular intervals. On each occasion, 10 tubes of each type were randomly selected and weighed on an analytical balance. 

#### 4.2.2. Draw Volume during the Time

Draw volume was measured in Ljubljana at an altitude of 300 m with a 5-mL Bang burette filled to the mark with Milli-Q water. The experimental technique was more precisely described elsewhere [21,22]. The nonreduced air pressure varied between 972 and 994 hPa, and the ambient temperature was (24 ± 1) °C.

### 4.3. Contaminants or Potential Additives

#### 4.3.1. Infrared Spectra

Infrared spectra were recorded on a Bruker FTIR Alpha Platinum ATR spectrometer (Billerica, MA, USA) in reflectance mode. Anticoagulant was extracted with Milli-Q water from the randomly selected tubes of the same brand. A composite sample was prepared and dried to constant mass at 50 °C under the nitrogen flux. Model solutions of sodium citrate, citric acid, citrate buffer, K_3_EDTA, and K_3_EDTA in the citrate matrix were treated equally. Anticoagulants extracted from the tubes and citrate model solutions formed a solid gel-type precipitate. Dried citric acid and K_3_EDTA were crystalline.

#### 4.3.2. Atomic Absorption Spectrometry

Potassium and magnesium concentrations in blood collection tubes were determined in solutions obtained by the addition of Milli-Q water in a nominal volume. The VARIAN AA240 Atomic Absorption Spectrometer was used. Experimental conditions are summarised in Table 9.

## 5. Conclusions

By introducing an easy-to-perform UV spectrometric method for citrate determination, we enabled for the first time a direct insight into the quality of citrate anticoagulant tubes before their intended use for specimen collection by using a purified water model. The approach reaches beyond the water loss evaluation during the time, draw-volume changes during shelf life, and contaminants determination, even though we include these aspects, too.

The major achievement is that by the complementarity use of the two methods of anticoagulant concentration determination and by using purified water as a model, we made possible the evaluation of the following:

- the accuracy of the anticoagulant amount added into the tubes by a producer;
- the accuracy of the volume of anticoagulant solution in the tube at the instant of examination;
- the anticoagulant concentrations for a nominal draw or draw volume.

The approach involves anticoagulant determination in a composite sample obtained by the anticoagulant water extraction from several tubes of the same type and dilution to a known volume with purified water on one side and anticoagulant determination in individual tubes after the addition of purified water in a volume corresponding to the nominal draw volume on the other.

We applied the developed methodology to the 105, 109, and 129 mmol/L citrate anticoagulant evacuated blood collection tubes. Nominal draw volumes ranged from 1.8 mL to 4.5 mL. Buffered and unbuffered citrate tubes were involved. The examined brands were Vacuette^®^, Greiner BIO-ONE, Vacutube, LT Burnik d.o.o., and BD Vacutainer^®^.

The results confirmed that all the producers control the draw volume well and that it mostly remains within the 10% tolerance limit during shelf life. For Vacutube, water loss during the time was observed. Contamination with potassium or magnesium or both was noticed for all the examined tubes, but the values for composite samples did not exceed 0.21 mmol/L.

Other aspects of quality that were until now not possible to control by the end user reveal the complexity of the citrate anticoagulant evacuated blood-collection tubes’ production. The anticoagulant amount added into the tubes during production had a relative bias between 3.2 and 23.0%. The anticoagulant volume deficiency at the instant of examination expressed as a relative bias ranged between −11.6 and −91.1%. The anticoagulant concentration relative bias after the addition of purified water in a volume that equalled a nominal draw volume extended from 9.3 to 25.7%. For all three examined parameters, the 10% tolerance limits were exceeded.

The suggested methodology enables medical laboratories to gain better insight into the characteristics of the citrate blood collection tubes as one of the preanalytical variables. In situations that require anticoagulant adjustment for accurate results of coagulation tests, this can help make the right decisions. The methodology gives producers additional means of controlling the quality of their production process.

## Figures and Tables

**Table 1:** Within-day repeatability of the spectrometric procedure tested on the 14.12 mmol/L trisodium citrate stock standard model solution; $\bar{c}\pm s=\left(1.415_{0}\pm 0.004_{3}\right)\;\mathrm{mmol}/L,\;n=10$.

	1	2	3	4	5	6	7	8	9	10
*A* _210 nm_	0.28716	0.28839	0.28825	0.28799	0.28860	0.28827	0.28647	0.28802	0.28620	0.28868
*c* (mmol/L)	1.4118	1.4178	1.4171	1.4159	1.4189	1.4173	1.4085	1.4160	1.4071	1.4192

**Table 2:** Within-laboratory reproducibility of the six-point (*n* = 6) calibration function (*y* = *a*·*x* + *b*) during a six-month period.

Date	*a*	*s_a_*	*b*	*s_b_*	*s_y_*_/*x*_	*R* ^2^
21 February 2022	0.1992	0.0041	0.0146	0.0091	0.0135	0.99835
25 February 2022	0.2032	0.0010	0.0002	0.0021	0.0032	0.99991
28 February 2022	0.2039	0.0010	0.0006	0.0023	0.0034	0.99990
11 March 2022	0.2021	0.0006	−0.0004	0.0013	0.0019	0.99997
6 April 2022	0.2027	0.0013	0.0022	0.0029	0.0043	0.99984
5 May 2022	0.2032	0.0028	0.0055	0.0064	0.0094	0.99922
16 June 2022	0.2038	0.0008	−0.0009	0.0017	0.0026	0.99994
7 July 2022	0.2021	0.0005	0.0018	0.0011	0.0016	0.99998
17 August 2022	0.2050	0.0008	−0.0047	0.0018	0.0027	0.99994

**Table 3:** Minimal and maximal citrate concentrations determined on a particular day in the blood collection tubes on the Milli-Q water model and their associated combined standard uncertainties.

(mmol/L)	17 August 2022	7 July 2022	16 June 2022	5 May 2022	6 April 2022	11 March 2022	28 February 2022	25 February 2022	21 February 2022
*c*_min_	11.9	11.7	11.6	11.6	11.6	11.7	11.5	13.7	14.1
*u*_c_	±0.2	±0.1	±0.2	±0.5	±0.3	±0.1	±0.2	±0.2	±0.7
*c*_max_	17.7	15.8	16.3	16.4	16.0	15.7	14.7	15.9	17.4
*u*_c_	±0.2	±0.2	±0.2	±0.5	±0.3	±0.2	±0.2	±0.2	±0.7

**Table 4:** Results of the ANOVA test for the tubes of producer B, the asterisks indicate which between-groups differences proved significant.

**B_1.8_31.7.**	**Count**	**Sum**	**Average**	**Variance**	**Differences**				
25.02.	10	64.5799	6.45799	0.000517959	*				
11.03.	10	64.3702	6.43702	0.000437162		*			
06.04.	10	64.1965	6.41965	0.000752418		*			
5.05.	10	63.8514	6.38514	0.000254645			*		
16.06.	10	63.4715	6.34715	0.000271512				*	
7.07.	10	63.4062	6.34062	0.000507422				*	
**B_3.6_31.8.**	**Count**	**Sum**	**Average**	**Variance**	**Differences**				
25.02.	10	66.8741	6.68741	0.000588828	*				
11.03.	10	66.6654	6.66654	0.000599349		*			
06.04.	10	66.3714	6.63714	0.000796518			*		
5.05.	10	66.1818	6.61818	0.000428091			*		
16.06.	10	65.8877	6.58877	0.00035316				*	
7.07.	10	65.5968	6.55968	0.000334517					*

* In contrast to tubes A and C, tubes B lost water during the four-month period.

**Table 5:** Results of citrate determination in composite samples prepared from the blood collection tubes with Milli-Q water and expressed as an anticoagulant amount per tube (*n*_ac_dtmn_) and the expected anticoagulant concentration (*c*__*V*_total_nom_) if the nominal draw-volume and nominal anticoagulant volume are both compliant with the values declared by the producer.

Tubes	*c*_ac_nom_ (mmol/L)	*n*_ac_expt_ (μmol)	*n*_ac_dtmn_ (μmol)	*n*_ac_ Relative Bias (%)	*c*__*V*_total_nom_ (mmol/L)
A_1.8_9.4.	109	21.8	26.3	20.6	13.2
A_1.8_9.7.	109	21.8	26.8	23.0	13.4
B_1.8_31.7.	109	21.8	23.6	8.2	11.8
C_1.8_31.3.	109	21.8	26.8	22.8 *	13.4
C_2.7_31.5.	109	32.7	38.4	17.4	12.8
B_3.6_31.8.	129	51.6	56.3	9.0	14.1
C_4.5_31.7.	105	52.5	55.0	4.8	11.0
C_4.5_31.12.	105	52.5	56.1	6.9	11.2
C_4.5_30.6.	129	64.5	66.6	3.2	13.3

* Irregularities in the absorption spectrum.

**Table 6:** Results of citrate determination (*c*_ac_dtmn_) after Milli-Q water addition into blood collection tubes (*n* = 10) in a volume that equals the nominal draw volume (*V*__draw_nom_) and other associated or derived parameters.

Tubes ^1^	*V*__draw_nom_ (mL)	*c*_ac_expt_ (mmol/L)	*c*_ac_dtmn_ (μmol)	*c*_ac_ Relative Bias (%)	*V*_ac_nom_ (mL)	*V*_ac_ (mL)	*V*_ac_ Relative Bias (%)
A_1.8_9.4.	1.8	10.9	13.6	24.8	0.2	0.134	−33.1
A_1.8_9.7.	1.8	10.9	13.7	25.7	0.2	0.157	−21.6
B_1.8_31.7.	1.8	10.9	13.0	19.0	0.2	0.018	−91.1
C_1.8_31.3.	1.8	10.9	14.8	35.8 *	0.2	0.008	−95.9
C_2.7_31.5.	2.7	10.9	13.4	22.9	0.3	0.164	−45.3
B_3.6_31.8.	3.6	12.9	14.3	10.9	0.4	0.334	−16.6
C_4.5_31.7.	4.5	10.5	12.1	15.2	0.5	0.048	−90.4
C_4.5_31.12.	4.5	10.5	12.1	15.2	0.5	0.137	−72.6
C_4.5_30.6.	4.5	12.9	14.1	9.3	0.5	0.223	−55.5

^1^ Evaluated on the 21st of March 2022. * Irregularities in the absorption spectrum.

**Table 7:** Potassium and magnesium concentrations determined in a composite sample prepared from the blood collection tubes of a particular type with Milli-Q water and expressed as expected if the *V*__draw_nom_ and *V*_ac_nom_ are compliant with the declaration.

Tubes	*c*_ac_nom_ (mmol/L)	Expiration Date	*V*__draw_nom_ (mL)	*V*_ac_nom_ (mL)	*c* (K^+^) (μmol/L)	*s* (*n* = 3)	*c* (Mg^2+^) (μmol/L)	*s* (*n* = 3)
A_1.8_NR	109	3 March 2022	1.8	0.2	/	/	209.1	±1.8
A_1.8	109	9 April 2022	1.8	0.2	/	/	187.0	±1.9
B_1.8	109	31 December 2021	1.8	0.2	/	/	208.3	±1.4
B_3.6_129	129	28 February 2022	3.6	0.4	/	/	46.4	±0.1
C_1.8	109	30 September 2021	1.8	0.2	139.3	±2.6	/	/
C_2.7	109	30 November 2021	2.7	0.3	146.8	±1.9	/	/
C_4.5_105	105	31 July 2022	4.5	0.5	170.2	±0.7	167.5	±0.4
C_4.5_129	129	30 June 2022	4.5	0.5	168.4	±3.8	164.1	±0.2

**Table 8:** Characteristics of the examined evacuated citrate or buffered citrate blood-collection tubes.

Abbreviation	Anticoagulant *c* (mmol/L)	Expiration Date	Draw Volume (mL)
B_1.8	109	31 December 2021	1.8
C_1.8	109 *	30 September 2021	1.8
C_2.7	109 *	30 November 2021	2.7
A_1.8_9.4.	109	9 April 2022	1.8
A_1.8_3.3._NR	109	3 March 2022	1.8
A_1.8_14.4._NR	109	14 April 2022	1.8
A_1.8_9.7.	109	9 July 2022	1.8
B_1.8_31.7.	109	31 July 2022	1.8
B_3.6_31.8.	129	31 August 2022	3.6
C_1.8_31.3.	109 *	31 March 2022	1.8
C-2.7_31.5.	109 *	31 May 2022	2.7
C_4.5_30.6.	129 *	30 June 2022	4.5
C_4.5_31.7.	105 *	31 July 2022	4.5
C_4.5_31.12	105 *	31 December 2022	4.5

* Buffered trisodium citrate.

**Table 9:** Experimental conditions for the determination of potassium and magnesium with atomic absorption spectrometry.

Metal Ion	K^+^	Mg^2+^
Wavelength (nm)	766.5	285.2
Slot width (nm)	1	0.5
Concentration range (mg/L)	0.25 to 1	0.05 to 05
Medium	1% HCl	1% HCl

## Data Availability

Data are contained within the article.

## Sample Availability

Samples of the compounds are not available from the authors.
